# Does measuring early basal serum follicular lutinising hormone assist in predicting In vitro fertilization (IVF)/Intracytoplasmic sperm injection (ICSI) outcome?

**DOI:** 10.1186/1477-7827-5-32

**Published:** 2007-07-20

**Authors:** Ahmed Kassab, Luca Sabatini, Gidon Lieberman, Amanda Tozer, Ariel Zosmer, Colin Davis, Talha Al-Shawaf

**Affiliations:** 1 Barts and The London Centre for Reproductive Medicine, Kenton and Lucas Block, St Bartholomew's Hospital, Barts and The London NHS Trust, West Smithfield, London EC1A 7BE, UK

## Abstract

**Background:**

The aim was to examine the correlation of early follicular serum lutinising hormone (LH) and the clinical outcome of assisted reproduction technique (ART).

**Methods:**

An observational study included 1333 consecutive women undergoing invitro fertilization (IVF) and intracytoplasmic sperm injection (ICSI). 964 women were having their first cycle of ART. Data were entered prospectively. All women had serum LH measured in the 6 months before the index cycle studied. No repeat cycles were included. The main outcomes measured were clinical pregnancy (CP) and live birth (LB) correlation to serum LH. Forward multivariate stepwise regression analysis was applied, and other statistical tests were used as appropriate.

**Results:**

There was non significant correlation between basal serum LH and CP and LB in the polycystic ovary syndrome group (R2 = 0.02, F = 1.7 and P = 0.76) (R2 = 0.01, F = 2.6 and P = 0.77) respectively after adjusting for age, BMI, day of oocyte retrieval, starting dose, total dose of stimulation, type of gonadotrophin used, number of oocytes retrieved, fertilization rate and number of embryos transferred. Other aetiological causes group there was similarly non significant correlation between basal serum LH and CP (R2 = 0.05, F = 13.1 and P = 0.66), nor for LB (R2 = 0.007, F = 4.5 and P = 0.9).

**Conclusion:**

Early follicular serum LH measurements in the 6 months before IVF/ICSI treatment cycle did not correlate with the clinical pregnancy or the live birth rate.

## Background

Controversy and debate still exist in defining ovarian reserve, methods of testing, and its value in general fertility assessment and in assisted reproduction technology (ART) [1-3].

Age is the most consistent variable that affects fertility potential. Other tests for measuring ovarian reserve or predicting outcome of fertility treatments have been inconsistent in their prediction [1,2]. Basal (day 1–4 of the follicular phase menstrual cycle) serum follicular stimulating hormone (FSH) measurement, has been historically used as a predictor of ovarian reserve ART [4-6] because of lower cost and ease of measurement. Women with normal basal FSH and luteinising hormone (LH) levels and those with a high LH: FSH ratio behaves as "normal "and" high" responders respectively, and classically will have adequate number of mature oocytes available for fertilization following standard ovarian stimulation [6-8]. Patients with high FSH may respond poorly to standard ovarian stimulation both in terms of oocyte numbers and outcome of treatment [4,7].

Basal serum LH is measured as part of routine assessment of subfertile women prior to ART [9]. Till recently serum LH levels has been considered to be essential in the diagnosis of polycystic ovary syndrome (PCOS). The Rotterdam ESHRE/ASRM- Sponsored PCOS consensus workshop group has recommended removal of LH measurement from the new revised criteria for PCOS [10,11].

It was suggested that basal serum LH could predict ovarian response to controlled ovarian hyperstimulation (COH) [12-14]. Others did not confirm the value of measuring serum basal LH in determining ovarian reserve or the clinical outcome of ART [15]. The aim of this study is to determine the correlation between basal serum LH and clinical pregnancy (CP) and live birth (LB) of consecutive and first cycle IVF and ICSI.

## Materials and methods

Data from 1333 consecutive cycles of IVF ± ICSI performed in our centre were evaluated. The data were extracted from a computer database. Serum FSH, LH, and estradiol (E_2_) were measured in the early follicular phase (day 1–4) within six months of the start of each treatment cycle. As a rising serum E_2 _is associated with a drop in the pituitary FSH and LH levels [16], these hormones were reassessed on typically day 1 or 2 of the next cycle, when the serum E_2 _levels were above 200 pmol/L. When serum E_2 _level were <200 pmol/L, gonadotrophin levels were considered to represents a true basal value [4,16]. In cases of amenorrhea or oligomenorrhea withdrawal bleeding was induced using medroxy progesterone acetate 5 mg twice daily for five days followed by measuring serum FSH, LH and E_2 _on day 2–4.

The long down regulation GnRH agonist protocol was employed in all cycles. Luteal phase down regulation was used except in cases with oligomenorrhea and irregular cycles where the start day was on day two of cycle, of natural or induced period (follicular phase down regulation). Pituitary down-regulation was achieved by buserelin 300 micrograms nasal inhalation 8 hourly, nafarelin 400 micrograms nasal inhalation 12 hourly or buserelin 500 micrograms daily by subcutaneous injection. The dose was reduced by 50% when down regulation was confirmed. COH was achieved by daily injection of gonadotrophins commencing at least two weeks later, after confirming full down-regulation by means of the absence of follicular activity, endometrial proliferation <5 mm or cyst formation at baseline ultrasound scan.

The gonadotrophin, used for controlled ovarian hyperstimulation (COH) was recombinant FSH (r-FSH) or human menopausal gonadotrophin (HMG) depending on the availability and cost [17,18]. The variables used to determine the gonadotrophin-starting dose were age, body mass index (BMI), previous response to COH and basal serum FSH level [19]. Neither the basal serum LH nor the FSH: LH ratio was a variable to guide the COH. In brief, the dose used in the COH protocols was: 110 IU/day if < 30 years of age, 150 IU/day if 30–34 years of age, 225 IU/day if 35–38 years of age and 300 IU/day if the woman age is above 38. If the basal FSH level was >9.9 IU/L, the dose was increased by 50%. If BMI >30 (excluding polycystic ovary) the dose was increased by 50%.

Human chorionic gonadotrophin (hCG) 10,000 iu was administered when 3 or more follicles were greater than 18 mm in diameter and endometrial thickness of > 8 mm with triple line/grade B development and occasionally "A" grade. A cycle was abandoned if there were less than 3 follicles, ≥ 14 mm of diameter, seen on ultrasound after 10 to 12 days of gonadotrophin stimulation.

Oocyte retrieval was done under intravenous sedation using pethidine and midazolam according to the patient response. Povidone iodine in aqueous solution was used to clean vagina and then thoroughly washed with sterile water to clean all traces of the iodine. Aspiration was achieved using double lumen needle using hepernized saline as a flushing medium. Aspiration pump pressure was set around 100 mm Hg.

Oocytes were evaluated for evidence of normal fertilization (2 distinct pronuclei) 18 hours after insemination. Embryos of day 2 (4 cell stage) or day 3 (eight cell stage) of high quality were transferred. Grading was according to number of blastomeres and degree of fragmentation. Soft Wallace transfer catheter was used but if difficulties encountered to negotiate the internal cervical os it was changed to the stiffer Frydman catheter. Embryos were transferred on day 2 after oocyte retrieval, except after Friday's oocytes retrievals when they may be transferred on day 3.

Luteal support was given in the form of progesterone pessaries 400 mg 12 hourly from day of egg collection for 17 days. Pregnancy test performed 14 days after embryo transfer (ET). If the test is positive an ultrasound scan was arranged 2–3 weeks later to confirm viability and exclude multiple pregnancy. The use of progesterone pessaries continue in pregnant patients until eight weeks gestation.

Clinical pregnancy was defined as ultrasonographic confirmation of an intrauterine gestation sac with fetal heart movements four to five weeks following ET. A non-clinical pregnancy was defined as a positive serum or urine βhCG test, but no fetal heart activity detected on vaginal ultrasound. A miscarriage was defined as a CP that ended before 24 weeks. A live birth was defined as the delivery of at least one viable infant. Twins and triplets were counted as one LB.

Patient with hypothalamic hypogonadism and those undergoing COH for ovum donation program or embryo/oocyte cryopreservation prior to chemotherapy were not included in the study. The upper age limit for starting an IVF cycle in our centre was 45 years.

To study the effect of low basal serum LH (<3 IU/L) and high level (>8 IU/L), the study group was divided into 3 groups based on the basal serum LH level: Group I: ≤ 3 IU/L, Group II: 3.1–7.9 IU/L, Group III: ≥ 8 IU/L. These values were used to be consistent with previously published reports [12,13,20].

### Hormonal assay

Measurements were performed by a Bayer immuno-1 automated analyser (Bayer, Newbury, Bucks, UK). In our hospital, we measure the serum LH level by calibrating to the Second International Reference Preparation of the WHO (2^nd ^IRP 80/552). The sensitivity of the assay was 0.1 IU/L. The intra-assay and interassay coefficients of variation were 5.5% and 6.7% respectively. Serum LH range was 0.7 – 10 IU/L in the follicular phase.

### Outcome measures

The main outcome measures were CP rate and LB rate. Secondary outcome measured analysed included the total dose of gonadotrophins, number of days of stimulation, number of oocyte retrieved, fertilization rate, numbers of embryos available, number of cycles requiring coasting for ≥ 2 days, and miscarriage rate.

### Statistical analysis

A Kolmogorov-Smirnov test was used to assess all variables for normality. Since the distribution of LH levels was not parametric, comparison of LH levels in between the groups of the different outcome measures (CP, miscarriage and LB) was done using a Mann-Whitney *U *test. Comparison of number of oocytes, fertilization rate and number of embryos between the three groups of basal serum LH was calculated by Kruskal Wallis analysis of variance. When the overall effect was significant, multiple Mann Whitney *U *tests were used to determine the source of significance. For comparing nominal data, Chi square (χ^2^) test was performed with Yates correction where appropriate. A probability value (P value) < 0.05 was considered significant.

To diminish the effect of confounding variables the analysis was performed by aetiology specially considering the effect of those with PCOS and other aetiology group using forward multivariate stepwise regression analysis. Odds ratio (OR) and confidence interval (CI) were used whenever appropriate. The power analysis was 82.8% in detecting an LH difference of 2 IU/L assuming a population s = 12 IU/L. The a-error level was fixed at 0.05.

All statistical calculations were done using Microsoft Excel version 7 and SPSS (Statistical Package for the Social Science; SPSS Inc., Chicago, IL, USA).

## Results

A total of 1333 consecutive IVF ± ICSI cycles were included in the study. Table 1 and 2 describe the demographic characteristics of the women included. Total dose of gonadotrophin used ranged between 1000–8300 IU with median 2800 IU while total days of stimulation ranged between 9–23 days with a median of 15 days. Three embryos were transferred in 13% of cases, while 83% of cases had 2 embryos replaced in uterus and 4% had one embryo replaced.

**Table 1:** Demographic characteristics of study group

Number	1333
Age (years)	34 (18–45)
BMI	24 (19–37)
Basal LH level (IU/L)	5.3 (0.7–27.6)
Causes of infertility (%)	
Male	43
Tubal	21
Endometriosis	5.6
PCOS	9
Unexplained	21.4
Abandon cycles (%)	3.6

BMI = body mass index

Values of age, BMI and LH level are given as median (range).

**Table 2:** Characteristics of first cycle treatment

Number	964
Age (years)	33 (19–45)
BMI	25 (19–36)
Basal LH level (IU/L)	5.7 (0.7–27.6)
Causes of infertility (%)	
Male	47.2
Tubal	19.4
Endometriosis	6.7
PCOS	8.4
Unexplained	28.3
Abandon cycles (%)	4

BMI = body mass index

Value of age, BMI and LH level is given as median (range).

48 cycles (3.6%) did not go through oocyte retrieval due to low response with median basal serum LH 5.3 IU/L, which was not different from those who underwent oocyte retrieval 5.3 IU/L (P = 0.9). Nine hundreds and sixty four (72.3%) patients had first IVF/ICSI cycles. This subgroup basal serum LH ranged from 0.7 to 27.6 IU/L (median 5.2). Multivariable logistic regression anlysis of the entire study group shows that age (OR 0.942, 95% CI 0.917–0.968, P < 0.001) and basal serum FSH (OR 0.920,95% CI 0.876–0.965, P = 0.001) were significantly and independently related to whether or not there was pregnancy. Similar analysis showed that age (OR 0.940, 95% CI 0.914–0.968, P < 0.001) and basal serum FSH (OR 0.934,95% CI 0.887–0.984, P = 0.01) was significantly and independently related to whether or not there was a live birth following IVF and ICSI. Analysis of basal serum LH did not show a significant relation to pregnancy (OR 0.975, 95% CI 0.931–1.02, P = 0.267) nor to LB (OR 0.987, 95%CI 0.941–1.036. P = 0.594).

We separately analysed the PCOS group. The range of the basal serum LH in this subgroup was 1.5 to 27.6 (median 5.8). Forward multivariate stepwise regression analysis was performed to determine factors that were significantly and independently associated with variation in the clinical outcome. The dependant variable was the CP and LB. For the PCOS group there was non significant correlation between basal serum LH and CP (R^2 ^= 0.02, F = 1.7 and P= 0.76) or to LB (R^2 ^= 0.01, F = 2.6 and P = 0.77) after adjusting for age, BMI, day of oocyte retrieval, starting dose, total dose of stimulation, type of gonadotrophin used, number of oocytes retrieved, fertilization rate and number of embryos transferred. Applying the same principle for other aetiological causes group there was non significant correlation between basal serum LH and CP (R^2 ^= 0.05, F = 13.1 and P = 0.66), nor for LB (R^2 ^= 0.007, F = 4.5 and P = 0.9).

When the cycles were grouped by the basal serum LH range (group I ≤ 3 IU/L, group II 3.1–7.9 IU/L, and group III ≥ 8 IU/L) and applying the Kruskal-Wallis ANOVA test there was no statistical significance difference between the three groups in terms of CP rate (P = 0.4), miscarriage rate (P = 0.8) or LB rate (P = 0.2) (Table 3). Multiple Mann-Whitney U test showed that the cycle with highest number of oocytes, fertilization and embryos was in the group III.

**Table 3:** Ovarian stimulation response and IVF/ICSI outcome according to basal serum LH

	Oocytes retrieval cycle	Fertilization rate %	CP	Misc	LB
**Group I** LH = 3 IU/L	N = 160		N = 59	N = 6	N = 53
	10 (2–22)	56.3 (0–100)	36.8%	10.1%	33.1%
**Group II** LH 3.1–7.9 IU/L	N = 960		N = 291	N = 51	N = 240
	10 (0–38)	55.6 (0–100)	30.3%	17.5%	25%
**Group III** LH ≥ 8.0 IU/L	N = 213		N = 77	N = 16	N = 61
	11 (0–29)	56.3 (0–100)	36.2%	20.7%	28.6%
P value	<0.05	<0.05	NS	NS	NS

CP = clinical pregnancy, Misc = miscarriage, LB = live birth, N = number, NS = not significant

Values are given as median (range) or percentage.

P value based on Kruskal Wallis analysis of variance

## Discussion

In this study we have investigated the relationship between basal serum LH and ART outcome in a large number (1333) of consecutive and first (964) cycles IVF and ICSI cycles. Multivariate stepwise regression analysis identified that measuring basal serum LH did no correlate with the outcome of ART cycles in terms of CP rate, miscarriage rate or LB rate. But we have identified by multivariable regression analysis that younger women (age) and the lower the basal serum FSH, the more likely women are to have live birth following IVF/ICSI. When the cycles were grouped by serum LH values to identify if low and high levels influenced the CP, miscarriage or the LB we noticed no statistical difference in the clinical outcome. Jurema et al (2003) in their study on cycles using GnRH antagonists [15] found that baseline FSH and E_2_, but not LH were significantly lower in cycles with pregnancy and as a single hormone FSH demonstrated the highest accuracy for predicting IVF outcome in GnRH agonist cycles compared to LH or E_2_. Our analysis confirms that in cycles using GnRH agonist down regulation basal LH has no correlation to ART outcome.

Mukherjee et al. [13] suggested that an elevated day 3 FSH: LH ratio >3.6, in the presence of a normal day 3 FSH is predictive of a poor response to ovarian stimulation. In their study performed on 74 cycles, they suggested that a cycle day 3 serum LH value <3 IU/L was, predictive of a poor response to ovarian stimulation [13]. Similarly Noci et al. [12] stated that low basal serum LH values < 3 IU/L, predict reduced response to ovarian stimulation as judged by decrease peak E_2 _and a lower number of preovulatory follicles in ovulation induction cycles. It was speculated that when early follicular LH levels are low there may be reduced activity of one or more of the known ovarian regulators (i.e., steroids or proteins such as inhbin, activin, follistin or insulin-like growth factors), which can influence follicular growth through actions by autocrine or paracrine routes [12]. Later Noci et al. [20] updated their findings on 249 IVF cycles, when GnRH agonist desensitisation was used and stimulation with HP-FSH. They found no difference in ovarian response, but noted that high dose of FSH dose was required in those with serum LH <3 IU/L. However when we sub grouped our study population by serum LH levels similar to the above studies a statistical difference was found in cycles with basal serum LH ≥ 8 IU/L in relation to number of oocytes retrieved, fertilisation and total number of embryos available. Although this was statistically significant, the difference is of doubtful clinical value representing only an increase of one oocyte retrieved and one extra embryo available between those with <8 and those ≥ 8 IU/L. But essentially the main outcome measures of CP, miscarriage and LB were similar in between the groups. Others have reported that patients undergoing ART with low basal LH levels (< 3 IU/L) did not differ significantly from controls in terms of response to COH, but there was a clear trend toward poorer implantation and CP rates [14]. We have noticed the contrary, in that women with the lowest basal serum LH (<3 IU/L) have a tendency to higher LB, all be it not statistically significant.

Several explanations may be proposed for what we have observed. Although LH remains in the circulation after pituitary desensitisation following the use of GnRHa it is of low biological value, [21], but still can provide enough derive for adequate steriodogenesis in the developing follicles. It has been demonstrated that in a GnRHa down- regulated IVF patients, the response to FSH is independent of serum LH levels at the time of starting FSH administration or on the day of hCG administration [22,23]. A recent meta-analysis has confirmed that measuring serum LH during ovarian stimulation in ART cycles is at present of no value [24]. We measured basal serum LH between days 1–4 of non stimulated menstrual cycle as it coincides with measurements of serum FSH and E_2. _Measuring serum LH in a different day in the menstrual cycle especially in oligomenorrheic women may provide different results [25].

There is considerable debate about the value of supplementing LH in COH for ART. While van Wely et al. [18] in a meta-analysis concluded that the use of hMG resulted in higher CP rates than did the use of r-FSH in IVF/ICSI cycles after GnRH agonist down-regulation in a long protocol, Al-Inany et al. [17] showed no evidence of clinical superiority in CP rate for rFSH over different urinary-derived FSH gonadotrophins. They suggested additional factors should be considered when choosing a gonadotrophin regimen, including the cost, patient acceptability, safety and drug availability [18]. We have used during COH, hMG or r-FSH based on availability and cost as the evidence so far available indicate no difference in their efficacy.

Embryo quality has an important correlation with the outcome of IVF treatment [26] which we acknowledge. However, it remains subjective, with possible inner observer variation and does not provide means to identify unequivocally embryos with enhanced implantation potential. So to avoid the bias embryo grading was not included in the multivariate stepwise regression analysis.

Antral follicle count (AC) and serum anti müllarian hormone (AMH) have been suggested as more predictive of ART than basal FSH [27], but their predictive value is still not universally accepted [1]. Both these tests still require large studies and more stringent evaluation because of cost and need for more expert assessment and definition in regard to AC [1-3].

## Conclusion

This study shows that the basal serum LH has no useful predictive value for IVF/ICSI clinical pregnancy and live birth outcome. We suggest that serum LH measurement can be dropped from tests prior to ART and the measurement of other may be more predictive cost effective tests used. The value of measuring serum LH in routine fertility and endocrinological conditions remain to be confirmed by further studies.

## Competing interests

The author(s) declare that they have no competing interests.

## Authors' contributions

AK carried out collecting the data, participated in the sequence alignment, and drafted the manuscript. LS performed the statistical analysis. GL participated in the sequence alignment. AZ participated in the design of the study. AT and CD participated in design and coordination. TAS conceived the study, and participated in the design and drafted the manuscript. All authors read and approved the final manuscript.
